# Alcohol and tobacco consumption affects bacterial richness in oral cavity mucosa biofilms

**DOI:** 10.1186/s12866-014-0250-2

**Published:** 2014-10-03

**Authors:** Andrew Maltez Thomas, Frederico Omar Gleber-Netto, Gustavo Ribeiro Fernandes, Maria Amorim, Luisa Fernanda Barbosa, Ana Lúcia Noronha Francisco, Arthur Guerra de Andrade, João Carlos Setubal, Luiz Paulo Kowalski, Diana Noronha Nunes, Emmanuel Dias-Neto

**Affiliations:** Laboratory of Medical Genomics, Centro Internacional de Pesquisa, AC Camargo Cancer Center, São Paulo, SP Brazil; Curso de Pós-graduação em Oncologia, Fundação Antônio Prudente/AC Camargo Cancer Center, São Paulo, SP Brazil; Universidad de Los Andes, Bogota, Colombia; Department of Head and Neck Surgery and Otorhinolaryngology, AC Camargo Cancer Center and National Institute of Science and Technology in Oncogenomics (INCITO), São Paulo, SP Brazil; Department of Psychiatry, Universidade de São Paulo and of Faculdade de Medicina do ABC, São Paulo, SP Brazil; Departmento de Bioquímica, Instituto de Química, Universidade de São Paulo, São Paulo, SP Brazil; Virginia Bioinformatics Institute, Virginia Tech, Blacksburg, VA USA; Laboratory of Neurosciences (LIM27), Institute of Psychiatry, Faculdade de Medicina, Universidade de São Paulo, São Paulo, SP Brazil

**Keywords:** Alcohol, Tobacco, Microbiome, Oral cavity, 16S rRNA

## Abstract

**Background:**

Today there are more than 2 billion alcohol users and about 1.3 billion tobacco users worldwide. The chronic and heavy use of these two substances is at the heart of numerous diseases and may wreak havoc on the human oral microbiome. This study delves into the changes that alcohol and tobacco may cause on biofilms of the human oral microbiome. To do so, we used swabs to sample the oral biofilm of 22 subjects; including 9 control-individuals with no or very low consumption of alcohol and no consumption of tobacco, 7 who were chronic and heavy users of both substances and 6 active smokers that reported no significant alcohol consumption. DNA was extracted from swabs and the V1 region of the 16S rRNA gene was PCR amplified and sequenced using the Ion Torrent PGM platform, generating 3.7 million high quality reads. DNA sequences were clustered and OTUs were assigned using the *ARB SILVA database* and *Qiime*.

**Results:**

We found no differences in species diversity and evenness among the groups. However, we found a significant decrease in species richness in only smokers and in smokers/drinkers when compared to controls. We found that *Neisseria* abundance was significantly decreased in both groups when compared to controls. Smokers had significant increases in *Prevotella* and *Capnocytophaga* and reductions in *Granulicatella*, *Staphylococcus, Peptostreptococcus* and *Gemella* when compared to the two other groups. Controls showed higher abundance of *Aggregibacter*, whilst smokers/drinkers had lower abundances of *Fusobacteria*. Samples from only smokers clustered closer together than to controls and smokers/drinkers, and also had a significant reduction in inter-group dissimilarity distances, indicating a more homogenous group than controls.

**Conclusions:**

Our results indicate that the continued use of tobacco or alcohol plus tobacco significantly reduces bacterial richness, which apparently leads to a reduction in inter-group variability, turning the respective biofilms into a more homogenous microenvironment in terms of bacterial community composition, with possible consequences for human oral diseases.

**Electronic supplementary material:**

The online version of this article (doi:10.1186/s12866-014-0250-2) contains supplementary material, which is available to authorized users.

## Background

Each day nearly 4,000 people younger than 18 years old smoke their first cigarette, a quarter of which will become addicted [1,2]. Cigarette smoking has been linked to acute respiratory tract infections [3,4] and lung cancer [5], among other diseases [6] and on average, smokers die 10 years before non-smokers [7]. As a consequence, it is estimated that the annual number of tobacco-related deaths will grow from about 6 million today to about 8 million in 2030 [8]. The negative effects of alcohol ingestion are not far behind. Global prevalence rates of alcohol dependency or chronic alcohol use were estimated to range from 0-16% in 2002 [9]. A study estimated that the net effect of alcohol consumption on health was responsible for 3.8% of all global deaths and 4.6% of global disability-adjusted life-years [10]. Chronic alcohol use has been linked to liver cirrhosis, liver cancer and several other diseases [11]. In 2002, 1.3 billion people were estimated to have smoked tobacco, whilst 2 billion were estimated to have consumed alcohol [9]. Unfortunately, when combined, alcohol and tobacco have an even worse impact on health, and studies have shown that tobacco users are more prone to use alcohol and vice versa [12]. Together, these drugs account for 12% of worldwide mortality rates [9], creating a huge global economic burden [13–15], as they increase the risk of several diseases [9], including oral cancer [16].

The use of cigarettes by adults, as well as passive smoke exposure in children, has been associated with increased carriage of pathogenic organisms in the upper airways [17]. It has been reported that substances present in tobacco smoke alter the charge and other properties of oral epithelial cell surfaces, allowing the growth of certain pathogenic bacteria [18]. Cigarette smoke also affects the survival of specific bacterial species isolated from the oral cavity, inhibiting some species of *Neisseria* and gram-positive bacteria such as *Staphylococcus aureus* and *Streptococcus pneumonia* [19]. However, little is known about the effects of chronic use of alcohol and tobacco on the oral microbiome.

The human oral cavity harbors more than 700 different bacterial taxa, possessing relevant quantitative (microbial richness) and qualitative (microbial community composition) differences between individuals [20–22]. Also, oral microbial profiles tend to show patterns of relative intra-individual stability over time [23]. Bacterial communities differ between healthy and diseased oral cavities and, as they can cause or prevent infections, this may have a significant impact on general health [22,24]. As most of the microbiome cannot be grown *in vitro* [25,26], community profiling approaches based on the amplification of marker genes of bacterial DNA derived from samples of interest have become a popular way of surveying microbial communities. With the recent development of high-throughput sequencing technologies, the investigation of microbial communities using the 16S rRNA gene as a marker has become the standard method for profiling microbial communities in various ecological niches.

In the present study we investigate the effects of the chronic use of alcohol and tobacco over the oral microbiome, in terms of diversity and composition, using the 16S rRNA gene as a target and the Ion Torrent Personal Genome Machine (PGM) platform for high throughput sequencing. To our knowledge, this is the first large-scale study focused on evaluating the effects that chronic use of alcohol and tobacco have over the microbiome of human oral biofilms.

## Methods

### Subjects

A total of twenty-two subjects were recruited and signed an informed consent form prior to sample collection. A questionnaire was given to all subjects to evaluate general characteristics including oral health, oral hygiene habits, alcohol and tobacco use and others (Table 1). Subjects were examined by stomatologists and/or dentists and allocated to one of the following groups:

**Table 1:** **Subject characteristics**

**Demographics**	**C (n=9)**	**SD (n=7)**	**S (n=6)**	**P-Value**
Average age (years)	58.11 ± 8.28	59.86 ± 3.39	56.67 ± 2.49	0.8458
Gender	F:1/M:8	F:1/M:6	F:1/M:5	0.9522
Average height (m)	1.71 ± 0.09	1.72 ± 0.07	1.69 ± 0.02	0.6845
Average weight (kg)	80 ± 11.94	72.29 ± 4.99	79.66 ± 5.95	0.467
Average BMI	27.44 ± 4.11	24.26 ± 1.13	27.72 ± 1.49	0.2433
Average cigarettes/day	0	18.43 ± 4.07	24.5 ± 3.49	0.00022*
Average alcohol intake/day (mL) in the last 6 months	0.9 ± 0.7	41.3 ± 14.3	0.01 ± 0.01	0.004*
Estimated total drinks†	6.2 ± 2.7	1668.9 ± 598.8	0.44 ± 0.44	0.004*
Daily mouthwash use	Yes (1)	Yes (3)	Yes (1)	0.3132
	No (8)	No (4)	No (5)	
Tooth cleaning frequency (per day)	1× (0)	1× (1)	1× (1)	0.7669
	2× (4)	2× (3)	2× (2)	
	3× (5)	3× (3)	3× (3)	
Self reported gingival bleeding	Yes (4)	Yes (1)	Yes (3)	0.9676
	No (5)	No (6)	No (3)	
Use of dentures	Yes (4)	Yes (4)	Yes (3)	0.8858
	No (5)	No (3)	No (3)	
Frequency of dentist appointments	1×/year (5)	1×/year (2)	1×/year (1)	0.5425
	1×/2-5 years (1)	1×/2-5 years (2)	1×/2-5 years (1)	
	1×/5 years (2)	1×/5 years (1)	1×/5 years (2)	
	Never (1)	Never (2)	Never (2)	

Values are given as average ± standard error. Height is given in meters (m), weight in kilograms (kg) and average daily alcohol intake is shown in milliliters (mL) per day. † Estimated total drinks = (average alcohol intake/day (mL)) × (years drinking). C: controls; SD: smokers/drinkers; S: only smokers; F: females; M: males; BMI: body mass index. Statistically significant differences (p<0.05) are indicated (*) by the Kruskal-Wallis Test.

Smokers/Drinkers (SD) (n = 7): subjects belonging to this group were recruited at the Alcohol and Tobacco Abuse Group at the Institute and Department of Psychiatry, part of the São Paulo Medical School (FMUSP), in São Paulo, Brazil. All individuals in this group reported the use of at least 20 cigarettes/day with a regular smoking history of at least 10 years. These individuals also reported daily drinking habits (>1×/day, >3 drinks/occasion) and a regular drinking history of at least 10 years*.*

Controls (C) (n = 9): All subjects belonging to this group were healthy individuals recruited at the cancer screening campaigns at the AC Camargo Cancer Center, also in São Paulo, Brazil. Individuals from this group never smoked cigarettes or any other tobacco-related derivatives, reported no or very low consumption of alcohol, defined as: participants reporting the consumption of only low-ethanol beverages (<5% ethanol), in small doses (maximum of 1 drink/occasion), with a drinking history inferior to 3 years and absence of daily drinking during this period. None of these individuals used alcohol-containing mouthwash solutions.

Smokers (S) (n = 6): All subjects belonging to this group were recruited at the cancer screening campaigns at the AC Camargo Cancer Center. These individuals reported a regular smoking history of at least 20 cigarettes/day for the last 10 years and maintained their smoking habits prior to sample collection*.* Regarding ethanol consumption as an excluding aspect, the same criteria as the control group was adopted, including mouthwash use.

### Additional exclusion criteria for all groups

Additional exclusion criteria for all subjects were as follows: **1)** subjects diagnosed with cancer; **2)** subjects reporting the use of antibiotics within the last 3 months prior to sample collection; **3)** subjects reporting residence outside of the city of São Paulo in the past 5 years; **4)** presence of comorbidities such as HIV, diabetes and ischaemic heart disease; **5)** individuals younger than forty years of age and **6)** presence of malignant or potentially pre-malignant oral lesions, such as leukoplakia or erythroplakia.

This study was approved by the ethics in research committees of AC Camargo Cancer Center (protocol 1459/10) and University of São Paulo (protocol 0895/11).

### Sample collection and DNA extraction

In order to sample bacteria from the oral biofilm, we swabbed the following oral mucosal surfaces of each individual: the dorsum and the laterals of the tongue, the floor of the mouth and the buccal mucosa. Bacterial samples were collected from these areas using Sterile Foam Tipped Applicators and transferred to FTA elute cards, both from Whatman (Maidstone, UK), where DNA was preserved after chemical lysis of the cells. In order to obtain a better representation of the whole oral mucosa in each sample, we collected four perforations (7 mm^2^ each) from different areas of each card and then eluted each one in 30 μl of sterile water by incubating for 25 mins at 95°C, according to the manufacturer’s recommendations. These four elutions from a single subject were pooled together and used as template DNA for subsequent procedures.

### PCR amplification and sequencing of the V1-region of the 16S rRNA gene

#### PCR amplification

We amplified the eluted bacterial DNA using a previously described set of degenerated primers [27] which cover the V1-region of the 16S rRNA gene (F: 5′-AGAGTTTGATCMTGGCTCAG-3′ and R: 5′-TTACTCACCCGTICGCCRCT-3′), generating ~113 bp amplicons, compatible with the sequencing platform. For a better representation of the bacterial DNA, we performed a total of six simultaneous PCR amplifications for each subject. PCR mixes consisted of 2 μM of each primer, 5 μl of the 2X high-fidelity Taq DNA polymerase master-mix (Roche, Mannheim, Germany) and 2 μl of eluted DNA in a final volume of 10 μl. The cycling conditions included an initial denaturation step (94°C for 2 mins) followed by 35 cycles at 94°C for 15 secs and 60°C for 30 secs and a final extension step at 72°C for 5 mins.

#### Sequencing

We evaluated 2 μl of the amplification products in 8% silver-stained polyacrilamyde gels [28] and quantified the amplicons of each individual using a Qubit high-sensitivity double stranded DNA kit (Qiagen, Crawley, UK). The amplicons were purified using AMPure XP beads (Beckman Coulter, Indianapolis, USA), end-repaired and Ion Torrent adaptors with 10 bp barcodes were ligated to their ends using proprietary kits (Life Technologies, Carlsbad, USA). For emulsion PCR we pooled together equimolar amounts of amplicons from each sample, as indicated by a Bioanalyzer High Sensitivity chip (Agilent Technologies, Santa Clara, USA). All samples were sequenced on the Ion Torrent PGM platform (Life Technologies).

### Sequence analysis

#### Sequence filtering

Sequences processed by the latest version of the Ion Torrent server (version 3.6.2) were used as input into the *Qiime* (*Quantitative insights into microbial ecology*) software package (Version 1.6.0) [29]. We first removed sequences that had an average quality score < 20, then identified barcodes used for subject-assignment accepting no more than 2 mismatches and discarded sequences smaller than 30 nt or greater than 200 nt. PCR primers identified at the start or at the end of the reads, allowing a maximum of 1 mismatch, were trimmed. Sequences with no identifiable primers were discarded. After primer trimming we again removed all sequences under 30 nt. We used the remaining sequences for downstream analysis.

#### Sequence clustering and OTU filtering

After primer- and quality-filtering, we clustered the remaining sequences with 97% identity using UPARSE (implemented in USEARCH v7) [30], and picked the less dissimilar sequence of each cluster as its’ representative. Chimeric sequences (and clusters) were identified using UCHIME [31] and the Broad Institute’s chimera slayer database (version microbiomeutil-r20110519) and excluded. We then used the *Qiime* interface to perform BLAST [32] analysis (using default parameters, with a maximum e-value of 0.001) against the formatted SILVA 111 SSU database [33] for taxonomic rank assignment of each sequence and, subsequently, to each operational taxonomic unit (OTU). In order to decrease PCR- and sequencing-derived errors, OTUs that were not present in at least 25% of all samples, had less than three reads, or had no taxonomic classification, were discarded from further analysis.

#### Data normalization

To investigate differences in phyla and genera abundance between the three groups, raw counts were normalized using the following normalization method [34]:

$$ \mathrm{Normalized}\ \mathrm{count} = \mathrm{l}\mathrm{o}{\mathrm{g}}_{10}\left[\left(\mathrm{raw}\ \mathrm{count}/\mathrm{number}\ \mathrm{o}\mathrm{f}\ \mathrm{sequences}\ \mathrm{in}\ \mathrm{that}\ \mathrm{sample}\right) \times \mathrm{average}\ \mathrm{number}\ \mathrm{o}\mathrm{f}\ \mathrm{sequences}\ \mathrm{per}\ \mathrm{sample} + 1\right] $$

#### Alpha diversity

To investigate species diversity, richness and evenness, we used the same sequence sampling depth for all samples by rarefying the OTU table. Rarefaction curves, the Shannon-Weaver diversity index [35] and equitability for species evenness were calculated using *Qiime*. Observed species richness and richness estimators Chao1 [36] and ACE [37] were calculated using the R (Open sourced statistical software, Vienna, Austria - Version 3.0.1) Phyloseq package (Version 1.4.5) [38].

#### Beta diversity

The Euclidean distance matrix, the coordinates for the Principal Coordinate Analysis (PCoA), and the intra- and inter-dissimilarity indexes were calculated using *Qiime.*

### Statistical analysis

Kruskal-Wallis tests were performed to compare mean similarities between controls, smokers/drinkers and only smokers for phyla and genera log abundances. P-values were corrected for multiple testing [34] using: (t*p)/R; where t = total number of taxa tested; p = raw p-value and R = sorted rank of the taxon. Kruskal-Wallis tests were also performed on categorical data of the subject’s characteristics using R. Independent two tailed T tests were performed on different alpha diversity metrics between pairs of the three groups.

## Results and discussion

### Subjects

Among the 22 subjects studied here, 19 were males (8 controls, 6 smokers/drinkers and 5 only smokers) and 3 females (one in each group). The composition of the groups showed no statistical differences in terms of age, gender, weight and height. Whereas the self-reports of general oral health, oral hygiene habits, gingival bleeding, annual visits to the dentist and others were also similar among the groups, as expected, differences in alcohol and tobacco consumption showed to be statistically significant. Table 1 shows the general sample characteristics, including p-values to demonstrate, if present, statistical differences between the three groups. Two subjects, one belonging to the only smokers group and one to the smokers/drinkers group were positive for hepatitis C, whilst another subject from the smokers/drinkers group was hypertensive. As alpha and beta diversity analysis indicated that these samples were not significantly different from others in their respective group (data not shown), they were included in downstream analysis. With regards to the use of other drugs, only one subject that belonged to the only smokers group admitted to the use of cocaine but had not used the drug for over a year, and was therefore also included in the study.

### Sequence analysis

#### Sequence filtering

After size, quality and primer trimming, a total of 3,843,174 sequences remained, with an average of 174,689 sequences/sample and a mean sequence length of 72 ± 4.9 nt (standard deviation) after primer trimming and barcode removal.

#### Sequence clustering and OTU filtering

When all individuals were considered, a total of 2,931 clusters were obtained. Twenty-one (0.7%) of these were removed after being identified as chimeras by UCHIME, and 112 (3.8%) had no assigned taxonomy. After filtering to remove OTU clusters with less than 3 sequences and not present in at least 25% of all samples, 1,849 OTUs remained.

### Beta diversity between controls, only smokers and smokers/drinkers

#### Beta diversity

**Figure 1:**
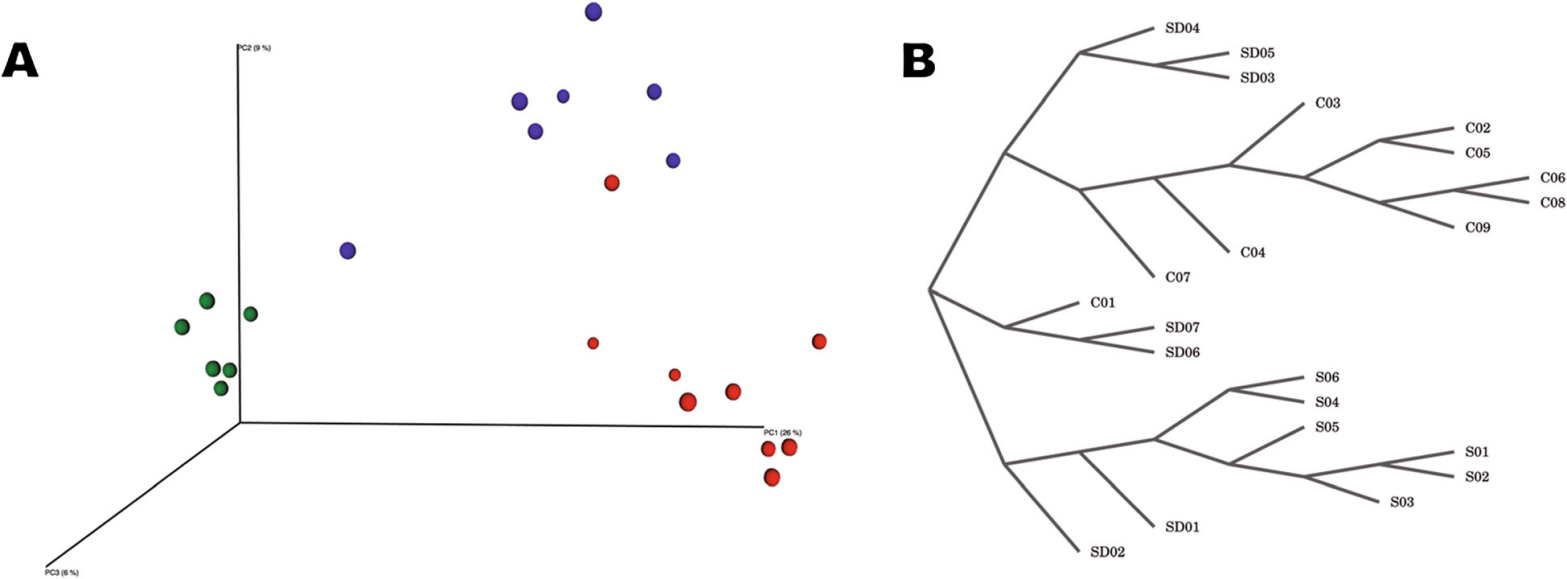
**Clustering of samples from C, S and SD groups using the binary Euclidean distance metric. (A)** The 3D PCoA plot shows the spatial distribution of samples of the groups: controls (C - red circles), smokers/drinkers (SD - blue circles) and only smokers (S - green circles) using the coordinates generated by the binary Euclidean distance metric. **(B)** A cladogram of all samples of the three groups produced by neighbor joining of the binary Euclidean distance matrix.

Using oral wash and broncheoalveolar lavage samples, a previous study found significant differences in bacterial communities when comparing smokers and non-smokers, suggesting that tobacco may significantly affect the oral microbiome [39]. In our study we first sought to evaluate if the 16S rRNA data generated would be capable of clustering individuals according to their use (or not) of alcohol/tobacco. We opted for the binary Euclidean distance metric due to the size of the 16S rRNA hypervariable region. Figure 1A shows the results of the PCoA plot using the distance matrix obtained by the binary Euclidean metric, demonstrating that 16S rRNA profiles clustered all individuals from the SD group together, as well as all samples from the S group. One of the control samples clustered closer to smokers/drinkers, whereas the other eight remaining control samples formed an independent group. Also, one of the smokers/drinkers samples clustered closer to only smokers, whilst the other six formed an independent group. The cladogram in Figure 1B indicates that all samples of the control group (except sample C01 which clustered closer to smokers/drinkers in the PCoA plot) belonged to the same main branch and that samples from smokers/drinkers were closer to only smokers than to controls. Also, all members of the only smokers-group clustered in the same tree branch. This consistent clustering suggests that alcohol and principally tobacco might influence the growth of certain bacterial species and may impact the normal abundance of bacteria commonly found in the oral cavity’s mucosa. It also suggests that smokers/drinkers have bacterial communities more similar to that of only smokers, rather than to controls.

### Intra- and inter-group similarity analyses

**Figure 2:**
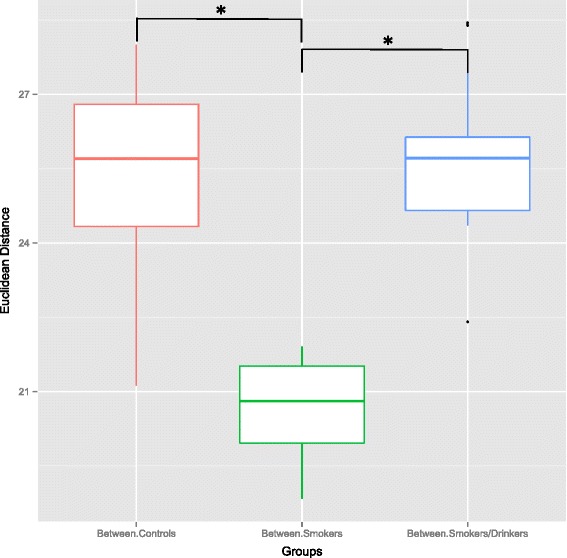
**Inter- and intra-group distances between C, S and SD groups.** The boxplot shows the average binary Euclidean distance when comparing intra and inter group dissimilarity. Significant comparisons between mean distances, as evidenced by a parametric *T*-test, are indicated (* = p < 0.05).

We then used the binary Euclidean metric to investigate if bacterial communities were more similar within each sample group or between groups, i.e. an intra-group *vs* inter-group dissimilarity analysis. Figure 2 illustrates our findings, demonstrating that samples from the only smokers group were statistically more similar, in terms of bacterial communities, between themselves than between samples from the two other groups. This suggests that tobacco may play a key role on quantitative/qualitative bacterial alterations, turning the only smokers sample group into the least dissimilar. Unfortunately we were not able to recruit a reliable group of alcohol-drinkers that do not use tobacco in order to properly address the role that ethanol alone would have over the oral mucosa’s microbiome. However, our analyses also demonstrate that the control group was the most dissimilar; evidencing how variable and diverse the healthy oral microbiome is between samples, especially in the absence of substances that appear to reduce the abundance of certain bacteria.

A recent study showed that even with the considerable intra- and interpersonal variation in the human microbiome, this variation could not be partitioned into community types that are predictive of each other, and were probably the result of life-history characteristics [40], which may include tobacco and/or alcohol consumption. Our results suggest that the chronic use of alcohol and principally tobacco imposes a constraint on the abundance of certain bacteria found in the oral cavity of subjects that do not ingest these substances on a regular and heavy basis, turning the otherwise healthy and diverse bacterial collection into a more homogeneous and restrict environment. Whereas it has been suggested that environment and diet do not significantly influence the composition of the oral microbiome [21], our data suggests that the consumption of alcohol and tobacco may be one of the primary determinants of microbial composition.

### Changes in alpha diversity caused by alcohol and tobacco

#### Species richness

**Figure 3:**
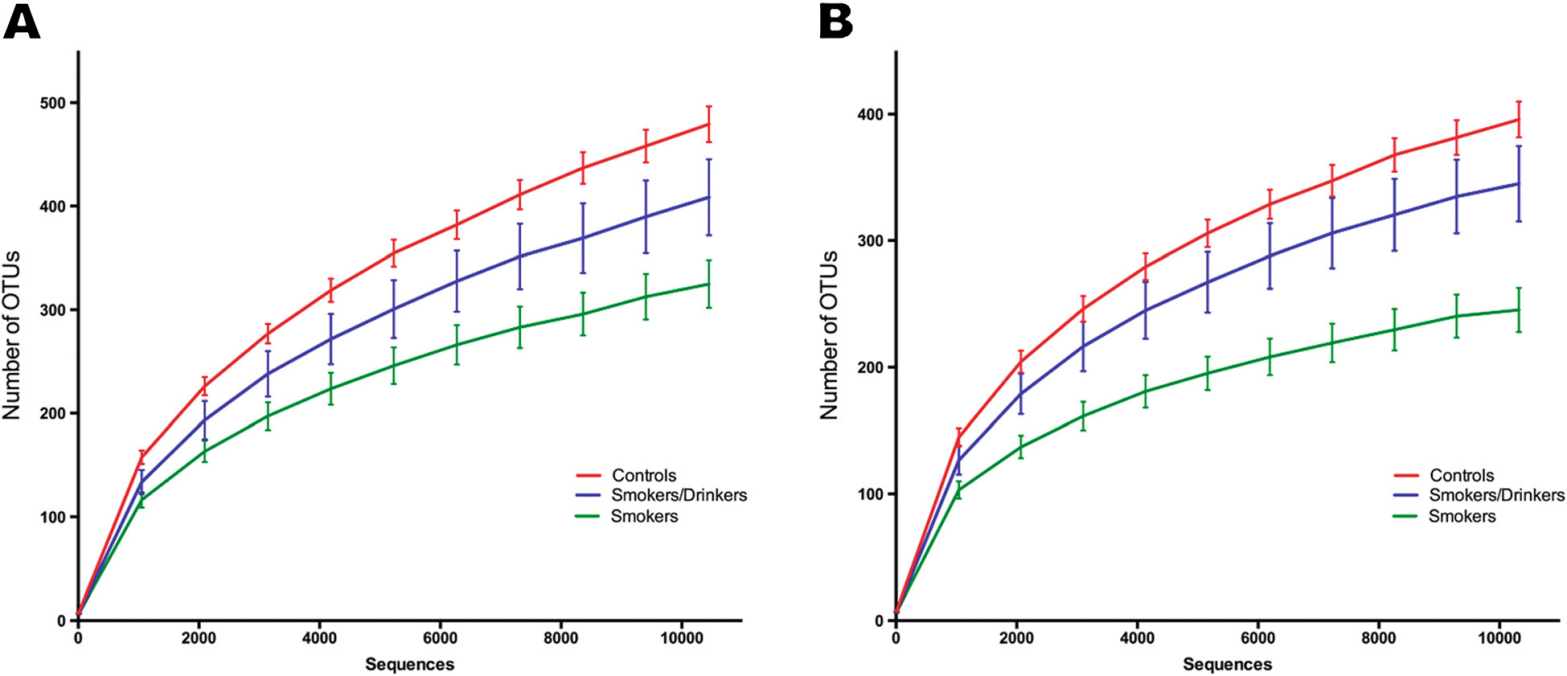
**Rarefaction curves at two different OTU abundance thresholds.** For each number of sequences the number of distinct OTUs was calculated; OTUs represented by one or two sequence reads were not considered. The distinct charts represent the rarefaction curves produced when we considered: **(A)** OTUs represented by at least 3 reads (or 0.03% of samples); **(B)** OTUs with a minimum of 100 reads (1%). Lines represent the average number of distinct OTUs ± standard error (S.E).

**Figure 4:**
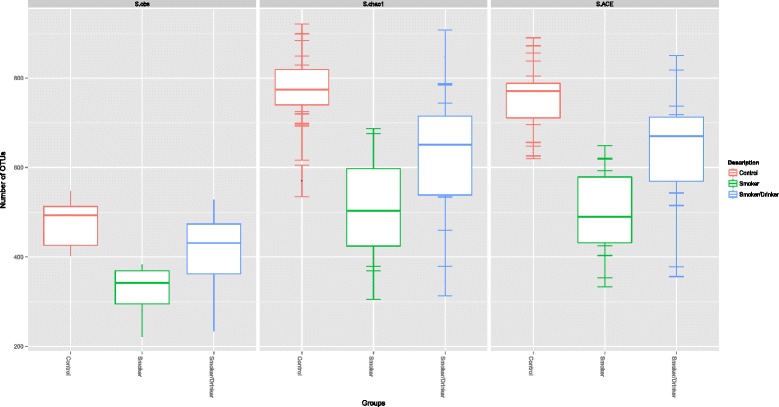
**Total species richness found in all three groups.** The figure shows the average total number of OTUs with at least 3 reads (0.03% of the individual samples) found for controls, only smokers and smokers/drinkers using three distinct richness indices: observed species metric (S.obs) and two richness estimators; chao1 (S.chao1) and ACE (S.ACE).

In order to measure species richness in the three groups we used the number of distinct OTUs observed in each sample when the same sequence sampling depth was applied at two different OTU-abundance thresholds (Figure 3). Saturation was not achieved when all OTUs represented by at least three reads were considered. This is likely to be derived from the combined effect of a still low coverage of very rare OTUs (those with frequencies below 0.03%, i.e. less than three reads/10,040 reads), together with PCR-amplification and/or sequencing artifacts. On the other hand, using this cutoff of at least three reads, which might underestimate very rare bacteria and precludes the representation of microorganisms non-amplifiable by this set of “universal primers”, as well as bacteria that cannot be discriminated on the basis of sequences derived from the variable region 1 (V1) of the 16S rRNA gene, we clearly see that the microbiome of all groups exceeds 300 distinct OTUs (Figure 3A), indicating a rich community. Indeed, bacterial communities of the oral cavity are one of the most complex across all body sites, second only to the colon [41]. When we considered a more stringent OTU abundance threshold (Figure 3B), a saturation trend for OTUs with at least 100 reads (1% of the sequences used for each subject) could be seen and indicates the presence of at least 200 OTUs with frequencies above this threshold (Figure 3B). When considering OTUs with at least three reads, we observed that the average species richness of controls was about 35% and 17% larger than that of only smokers and smokers/drinkers, respectively (Figure 3A-B). When the same sequencing depth was used to evaluate species richness between pairs of the three groups, using three distinct metrics, we observed a significant decrease in species richness for only smokers when compared to controls. A significant reduction in OTU richness between controls and smokers/drinkers was also found at the sampling depth of 142,161 reads. The reduction in richness (in both SD and S when compared to C) was observed when using the Observed Richness (only smokers p-value= 0.0005; smokers/drinkers p-value= 0.0446) and also for richness estimators Chao1 (only smokers p-value= 0.0013; smokers/drinkers p-value= 0.0465) and ACE (only smokers p-value= 0.0009; smokers/drinkers p-value= 0.0474) (Figure 4). Surprisingly, we found no statistical difference in species richness between only smokers and smokers/drinkers using any of these metrics (p > 0.05).

#### Species evenness

We evaluated species evenness through the use of equitability (mean **C**: 0.58; mean **SD**: 0.55; mean **S**: 0.55, where 1 indicates complete equitability) and found no statistical difference in species evenness between these three groups (p > 0.05) (Table 2). This suggests, for all groups, the presence of numerous species with low overall abundance and few highly abundant species. Data from the Human Microbiome Project showed that, within a cohort, bacterial members present in low abundance, low ubiquity or both, may contribute to high interpersonal variations of the microbiome [42], and this may apply here, by reducing microbial richness in only smokers and therefore reducing the dissimilarity between subjects of this group.

**Table 2:** **Alpha diversity measures for individuals according to the consumption of alcohol and/or tobacco**

**Samples**	**S.obs**	**S.chao1**	**S.ACE**	**Shannon-Weaver Index**	**Equitability**
C01	426	774	711	5.13	0.59
C02	546	845	873	5.49	0.6
C03	493	819	773	5.65	0.62
C04	403	570	641	4.71	0.54
C05	483	749	765	4.76	0.54
C06	494	740	771	5.33	0.6
C07	424	668	634	4.47	0.52
C08	513	777	788	6.01	0.66
C09	540	862	855	5.17	0.57
S01	340	522	494	4.64	0.56
S02	280	404	414	4.45	0.55
S03	223	342	343	3.63	0.47
S04	345	485	486	5.16	0.61
S05	377	622	634	4.8	0.56
S06	382	630	607	4.88	0.56
SD01	235	346	367	3.62	0.47
SD02	468	729	703	5.34	0.6
SD03	431	651	670	5.29	0.6
SD04	479	700	722	4.87	0.56
SD05	527	847	834	5.26	0.59
SD06	334	497	529	3.94	0.47
SD07	390	580	610	5.02	0.59

The table shows the number of OTUs with regards to species richness (observed, Chao1, ACE), species evenness (equitability) and diversity (Shannon-Weaver Index) for each sample using the sample sequence sampling depth (10,040 sequences).

#### Species diversity

Also, we evaluated species diversity using the Shannon-Weaver index and found no significant differences in diversity between each of the possible group pairs, but did see an increase in controls (mean **C**: 5.19, **SD**: 4.76 and **S**: 4.59). This suggests that the key difference in alpha diversity between controls, smokers/drinkers and only smokers lies solely in species richness. A previous study found a more diverse microbiome in the oropharynx of smokers than non-smokers [43], however it was restricted to tobacco-induced effects and no alcohol-drinking habits were reported. Another study evaluated the effects of chronic alcohol feeding on the intestinal microbiome of mice [44]. In this case, a reduction in species richness was observed along three time-points in alcohol-fed versus pellet-fed animals, and this decrease showed a negative correlation between species richness and the cumulative time mice were alcohol-fed. Another recent work [45] also found a reduction in species richness, this time in the oral biofilms covering the teeth of rats that received 30-days of a 20% ethanol-containing diet. Besides technical limitations due to the approach used for bacteria detection and quantification in the biofilms of rat teeth (restricted to probes for only 33 bacteria of the human oral microbiome) these authors were able to demonstrate a significant association between alcohol consumption and lower levels of bacterial richness. A reduction in microbial diversity has been associated with other diseases such as irritable bowel syndrome [46], eczema [47], Crohn’s disease [48], obesity [49] and recurrent *Clostridium difficile-*associated diarrhea [50], evidencing the crucial role that commensal bacteria play on keeping pathogenic microbes at bay and maintaining general homeostasis.

The chronic and simultaneous use of alcohol and tobacco appears to significantly decrease species richness in the human oral microbiome, which could lead to dysbiosis, the unbalancing of the relative abundance of individual components of the microbiota compared to their abundance in health [51]. It has been hypothesized that dysbiosis could lead to a variety of diseases [23]. Our data indicates that tobacco, together or not with alcohol, affects the microbiome leading to reduced species richness, through a mechanism that probably includes pro- and anti-inflammatory molecules [52]. To our knowledge, the work presented here is the first to evaluate microbial diversity and composition in biofilms of the oral mucosa in groups of human subjects with regards to the simultaneous and chronic use of alcohol and tobacco and the use of tobacco alone.

### Eubacterial phyla and genera most affected by alcohol and tobacco

#### Phyla log abundances

**Figure 5:**
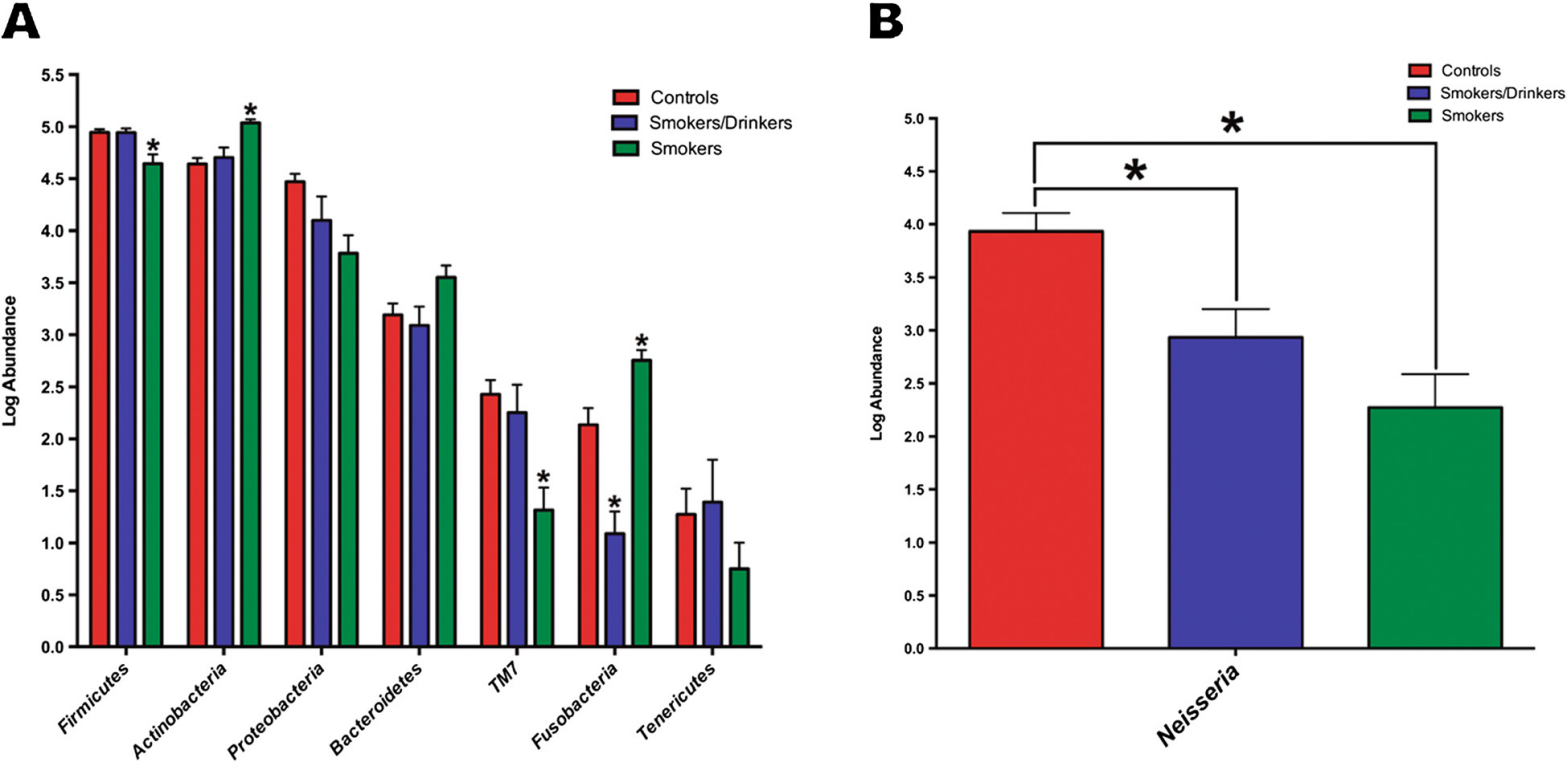
**Most abundant eubacterial phyla found in the three groups.** The figure represents the log abundance of **(A)** the most abundant bacterial phyla found in the oral mucosa, for the groups of controls, only smokers and smokers/drinkers; **(B)** *Neisseria* with a significant decrease in smokers/drinkers and only smokers when compared to controls. Columns represent the average log abundance ± standard error (S.E) and (*) indicates p < 0.05.

The most abundant phyla found in all groups were (in decreasing order of abundance): *Firmicutes*, *Actinobacteria*, *Proteobacteria*, *Bacteroidetes*, *TM7*, *Fusobacteria* and *Tenericutes* (Figure 5A). At the phylum level, six out of 16 phyla were found to have significant differential log abundances between the three groups (Additional file 1: Table S1). Only smokers had an increase of *Actinobacteria* and *Fusobacteria* and a decrease of *Firmicutes, Cyanobacteria, TM7* and *BD1-5* when compared to the two other groups, whilst smokers/drinkers had a decrease of *Fusobacteria. TM7* is a novel candidate bacterial division and previous studies have shown that microbes from this division are commonly found in the human oral flora at relatively low abundance, generally around 1%[53,54] and up to 8% [55] of the oral bacterial population, and a possible role of this phylum in periodontal disease has been previously suggested [53,56]. In only smokers, a significant reduction in log abundance of *Proteobacteria* could be observed (Kruskal-Wallis test, p=0.03), but after correcting for multiple taxa testing, this decrease was no longer significant (p=0.06). A reduction in the relative abundance of this phylum in broncheoalveolar-lavages of smokers when compared to non-smokers has been reported [39], pointing to a role that tobacco may play on reducing representatives of this phylum.

*Genera log abundances.* At the genus level, 47 out of the 279 (16.8%) identified genera were found to have significant differential log abundances between the three groups (Additional file 2: Table S2). The most abundant genera found in controls were (in decreasing order of abundance): *Veillonell*a, *Actinomyces*, *Streptococcus*, *Haemophilus* and *Neisseria*. In smokers/drinkers the most abundant genera were: *Veillonell*a, *Actinomyces, Rothia*, *Granulicatella* and *Streptococcus* and in only smokers were: *Actinomyces*, *Rothia*, *Veillonella*, *Streptococcus* and *Leucobacter*. All these genera have been reported before as common residents of the oral cavity and of oral biofilms [20,24,57]. We found a significant decrease in log abundance of *Neisseria* in both only smokers and smokers/drinkers when compared to controls (Kruskal-Wallis test p= 0.026; Figure 5B), but found no difference in the log abundance of this genus between SD and S. Whereas *Neisseria* is one of the most dominant genera found in the larynx, including laryngeal tumors [58], other studies have reported that the abundance of this genus is modulated by tobacco as its levels are reduced in the oral wash, oro-/nasopharyngeal swabs and bronchoscopic alveolar lavages of cigarette smokers [39,43,59].

Only smokers had significant increases in *Prevotella* and *Capnocytophaga* and decreases in *Granulicatella*, *Staphylococcus*, *Peptostreptococcus* and *Gemella* when compared to the other two groups. A significant decrease in abundance of *Peptostreptococcus* in smokers has been evidenced before [43], suggesting the susceptibility of this genus to smoke exposure. It is of interest to note that this particular reduction may be significant as several species belonging to this genus have shown to interfere in the growth of pathogenic bacteria in the upper respiratory tract [60]. Another genus that also seems to be modulated by smoking is *Gemella*, with a previous study also finding a decrease in the abundance of this genus [39]. In our analysis, the genus *Porphyromonas,* which has been shown to be increased in smokers [39,43] and to have a role in periodontitis [61,62]*,* was also found to have higher abundances in only smokers (mean **C**: 0.67, **SD**: 0.29 and **S**: 1.8). However, after multiple taxa correction, the p-value was no longer significant (Kruskal-Wallis test p-value=0.08). Subsequent reports using 16S rRNA sequence profiling of subgingival plaque identified an increase in several disease-associated organisms in smokers, including *Parvimonas*, *Fusobacterium*, *Campylobacter*, *Bacteroides*, *Dialister*, and *Treponema* spp. and a decrease in potential health-promoting taxa from the *Veillonella*, *Neisseria*, *Streptococcus*, and *Capnocytophaga* genera [63]. *Aggregibacter* levels were higher in controls, whilst smokers/drinkers had lower log abundances of *Fusobacteria* compared to C and S (Kruskal-Wallis test; p-values= 0.04417 and 0.02541, respectively).

## Conclusions

The study presented here has certain limitations that need to be considered. First we would like to point out that the sample size was relatively small (N=22) and limited to a set of individuals living in the city of São Paulo, Brazil. The study of larger cohorts, and a cohort composed of individuals that consume alcohol but do not smoke would be important for a better understanding of the individual effects of these substances. The study of individuals who have quit using alcohol and tobacco, as well as recent users, would be important to reveal how long a ‘normal’ microbiome persists and how long it takes to recover after the use of alcohol/tobacco. Besides the overall evaluation of oral hygiene habits and oral health status (Table 1), we did not check for the presence of periodontal disease. Our findings are also limited by the choice of PCR primers targeting the V1 region of the 16S rRNA gene. *In silico* analysis indicated that, whereas this primer pair covers about 54.5% of the eubacterial phyla present in the ARB SILVA database, certain phyla are clearly under-represented, including *Fusobacteria* and *Bacteroidetes*. Also, whereas the DNA extraction protocol used here does not include the use of physical lysis (such as a bead beating step), which may improve the representation of certain bacteria (especially Gram-negative), and whilst PCR/sequencing artifacts may still persist, all these effects should be homogeneous for all groups and should not greatly affect our conclusions. A previous study [64] reported that a correction for the number of rRNA operons influences the distribution of bacterial phyla found in oceans. Considering that a) this operon normalization correction was optimized for larger 16S rRNA amplicons and b) the authors suggest that this correction would not greatly affect phylum distributions in the human microbiome, this correction was not implemented here. A recently described tool, named CopyRighter [65] showed to be capable of improving estimates of relative OTU abundances in human microbiome profiles after rRNA copy-number correction, albeit we have not investigated whether the use of this tool would affect our main conclusions.

Taking these limitations into consideration, we found a reduced bacterial richness in both only smokers and smokers/drinkers when compared to controls. To our knowledge, no studies have evaluated, up to now, if alcohol and tobacco affect the microbial composition of the human oral mucosa. In this study we found that bacterial communities from subjects who chronically use tobacco – together or not with alcohol – cluster together and possess reduced species richness, suggesting that this substance may play a key role in reducing the oral cavity’s bacterial richness. This reduced richness might have contributed to the significant decrease in dissimilarity observed between subjects of the only smokers group, showing to be the most homogenous in terms of bacterial communities. Species diversity and species evenness were not significantly different between any of the group pairs. Although, independent of the metrics used, all analyses indicated a higher species-richness in controls.

Our data indicates the presence of over 300 distinct bacterial OTUs in the human oral mucosa (Figure 3A). Our rarefaction curves show that, despite the limitations of the primer pair used and the size of the amplicons, for each individual that was sampled, we were able to represent OTUs that had a frequency of at least 1%. At the phylum level, we found that 6 out of the 16 phyla were altered between individuals of the three groups, with only smokers possessing the greatest number of affected phyla. Only smokers had significant decreases in log abundance of *Firmicutes* and *TM7* among others and significant increases in *Fusobacteria* and *Actinobacteria*.

We found a significant decrease in *Neisseria* abundance in both only smokers and smokers/drinkers, reinforcing evidence that points to a modulation of this genus’s abundance by tobacco smoke. We found that two other genera, *Gemella* and *Peptostreptococcus,* were exclusively decreased in only smokers.

A recent study showed that modern dietary habits introduced in the early Neolithic period, including carbohydrate ingestion, affected the oral microbiome composition and diversity [66]. The authors conclude that the modern oral environment is likely to be less resilient to perturbations due to infection by pathogenic bacterial species or to dietary imbalances. Whereas the modern carbohydrate-rich diet appears to lead to a less diverse oral microbiome when compared to the hunter-gatherer diets, our results indicate further microbiome perturbations led by alcohol and tobacco consumption. The evidences presented here suggest that microbial richness preservation- and restoration-strategies may represent effective novel approaches for the prevention and treatment of some aspects of alcohol and tobacco related diseases.

### Availability of supporting data

Nucleic acid sequences are available at NCBI/GenBank/SRA under accession number “SRP044345”.

## Additional files

Additional file 1: Table S1. Phyla log abundance. Additional file 2: Table S2. Differential genera log abundance.

## Abbreviations

C: Controls
SD: Smokers/drinkers
S: Only smokers
PCoA: Principal coordinate analysis
OTU: Operational taxonomic unit
nt: Nucleotide
bp: Base pair
PCR: Polymerase chain reaction

## References

[1] Substance Abuse and Mental Health Administration (2013). Results from the 2010 National Survey on Drug Use and Health: National Findings.

[2] U.S. Department of Health and Human Services (2012). Preventing Tobacco Use Among Youth and Young Adults: A Report of the Surgeon General.

[3] Aronson MD, Weiss ST, Ben RL, Komaroff AL (1982). Association between cigarette smoking and acute respiratory tract illness in young adults. JAMA.

[4] Turkeltaub PC, Gergen PJ (1991). Prevalence of upper and lower respiratory conditions in the US population by social and environmental factors: data from the second National Health and Nutrition Examination Survey, 1976 to 1980 (NHANES II). Ann Allergy.

[5] Boyle P, Maisonneuve P (1995). Lung cancer and tobacco smoking. Lung Cancer.

[6] Fagerström K (2002). The epidemiology of smoking. Drugs.

[7] Jha P, Ramasundarahettige C, Landsman V, Rostron B, Thun M, Anderson RN, McAfee T, Peto R (2013). 21st century hazards of smoking and benefits of cessation in the united states. N Engl J Med.

[8] The Lancet (2013). Tobacco control: when economics trumps health. Lancet.

[9] **World Health Organization, Substance Abuse, Global Burden of Disease.** *ᅟ* 2002, **ᅟ:**ᅟ. Available: http://www.who.int/substance_abuse/facts/global_burden. Accessed 30 July 2013.

[10] Rehm J, Mathers C, Popova S, Thavorncharoensap M, Teerawattananon Y, Patra J (2009). Global burden of disease and injury and economic cost attributable to alcohol use and alcohol-use disorders. Lancet.

[11] Rhem J, Shield KD (2013). Global alcohol-attributable deaths from cancer, liver cirrhosis, and injury in 2010. Alcohol Res.

[12] Bobo JK, Husten C (2000). Sociocultural influences on smoking and drinking. Alcohol Res Health.

[13] Cummings KM, Stiles J, Mahoney MC, Sciandra R (1992). Health and economic impact of cigarette smoking in New York State, 1987–1989. N Y State J Med.

[14] Oh IH, Yoon SJ, Yoon TY, Choi JM, Choe BK, Kim EJ, Kim YA, Seo HY, Park YH (2012). Health and economic burden of major cancers due to smoking in Korea. Asian Pac J Cancer Prev.

[15] Cai L, Cu W, He J, Wu X (2014). The economic burden of smoking and secondhand smoke exposure in rural South-West China. J Asthma.

[16] Soerjomataram I, deVries E, Pukkala E, Coebergh JW (2007). Excess of cancers in Europe: a study of eleven major cancers amenable to lifestyle change. Int J Cancer.

[17] Brook I, Gober AE (2008). Recovery of potential pathogens in the nasopharynx of healthy and otitis media-prone children and their smoking and nonsmoking parents. Ann Otol Rhinol Laryngol.

[18] El Ahmer OR, Essery SD, Saadi AT, Raza MW, Ogilvie MM, Weir DM, Blackwell CC (1999). The effect of cigarette smoke on adherence of respiratory pathogens to buccal epithelial cells. FEMS Immunol Med Microbiol.

[19] Ertel A, Eng R, Smith SM (1991). The differential effect of cigarette smoke on the growth of bacteria found in humans. Chest.

[20] Paster BJ, Olsen I, Aas JA, Dewhirst FE (2006). The breadth of bacterial diversity in the human periodontal pocket and other oral sites. Periodontol.

[21] Nasidze I, Li J, Quinque D, Tang K, Stoneking M (2009). Global diversity in the human salivary microbiome. Genome Res.

[22] Lazarevic V, Whiteson K, Hernandez D, Francois P, Schrenzel J (2010). Study of inter- and intra-individual variations in the salivary microbiota. BMC Genomics.

[23] Costello EK, Lauber CL, Hamady M, Fierer N, Gordon JI, Knight R (2009). Bacterial community variation in human body habitats across space and time. Science.

[24] Aas JA, Paster BJ, Stokes LN, Olsen I, Dewhirst FE (2005). Defining the normal bacterial flora of the oral cavity. J Clin Microbiol.

[25] Pei Z, Bini EJ, Yang L, Zhou M, Francois F, Blaser MJ (2004). Bacterial biota in the human distal esophagus. PNAS USA.

[26] Zhou X, Bent SJ, Schneider MG, Davis CC, Islam MR, Forney LJ (2004). Characterization of vaginal microbial communities in adult healthy women using cultivation-independent methods. Microbiology.

[27] Sundquist A, Bigdeli S, Jalili R, Druzin ML, Waller S, Pullen KM, El-Sayed YY, Taslimi MM, Batzoglou S, Ronaghi M (2007). Bacterial flora-typing with targeted, chip-based Pyrosequencing. BMC Microbiol.

[28] Sanguinetti CJ, Dias-Neto E, Simpson AJ (2004). Rapid silver staining and recovery of PCR products separated on polyacrylamide gels. Biotechniques.

[29] Caporaso JG, Kuczynski J, Stombaugh J, Bittinger K, Bushman FD, Costello EK, Fierer N, Peña AG, Goodrich JK, Gordon JI, Huttley GA, Kelley ST, Knights D, Koenig JE, Ley RE, Lozupone CA, McDonald D, Muegge BD, Pirrung M, Reeder J, Sevinsky JR, Turnbaugh PJ, Walters WA, Widmann J, Yatsunenko T, Zaneveld J, Knight R (2010). QIIME allows analysis of high-throughput community sequencing data. Nat Methods.

[30] Edgar RC (2013). UPARSE: Highly accurate OTU sequences from microbial amplicon reads. Nat Methods.

[31] Edgar RC, Haas BJ, Clemente JC, Quince C, Knight R: **UCHIME improves sensitivity and speed of chimera detection.** *Bioinformatics* 2011, **ᅟ:**ᅟ. doi:10.1093/bioinformatics/btr38. 10.1093/bioinformatics/btr381 PMC3150044 21700674

[32] Altschul SF, Gish W, Miller W, Myers EW, Lipman DJ (1990). Basic local alignment search tool. J Mol Biol.

[33] Quast C, Pruesse E, Yilmaz P, Gerken J, Schweer T, Yarza P, Peplies J, Glöckner FO (2013). The SILVA ribosomal RNA gene database project: improved data processing and web-based tools. Nucleic Acids Res.

[34] Sanapareddy N, Legge RM, Jovov B, McCoy A, Burcal L, Araujo-Perez F, Randall TA, Galanko J, Benson A, Sandler RS, Rawls JF, Abdo Z, Fodor AA, Keku TO (2012). Increased rectal microbial richness is associated with the presence of colorectal adenomas in humans. ISME J.

[35] Shannon CE, Weaver W (1949). The mathematical theory of communication. AT&T Tech J.

[36] Chao A (1987). Estimating the population size for capture-recapture data with unequal catchability. Biometrics.

[37] Chao A, Lee SM (1992). Estimating the number of classes via sample coverage. J Am Stat Assoc.

[38] McMurdie PJ, Holmes S (2013). phyloseq: An R package for reproducible interactive analysis and graphics of microbiome census data. PLoS One.

[39] Morris A, Beck JM, Schloss PD, Campbell TB, Crothers K, Curtis JL, Flores SC, Fontenot AP, Ghedin E, Huang L, Jablonski K, Kleerup E, Lynch SV, Sodergren E, Twigg H, Young VB, Bassis CM, Venkataraman A, Schmidt TM, Weinstock GM (2013). Comparison of the respiratory microbiome in healthy nonsmokers and smokers. Am J Respir Crit Care Med.

[40] Ding T, Schloss PD (2014). Dynamics and associations of microbial community types across the human body. Nature.

[41] Human Microbiome Project Consortium (2012). Structure, function and diversity of the healthy human microbiome. Nature.

[42] Li K, Bihan M, Methé BA (2013). Analyses of the stability and core taxonomic memberships of the human microbiome. PLoS One.

[43] Charlson ES, Chen J, Custers-Allen R, Bittinger K, Li H, Sinha R, Hwang J, Bushman FD, Collman RG (2010). Disordered microbial communities in the upper respiratory tract of cigarette smokers. PLoS One.

[44] Bull-Otterson L, Feng W, Kirpich I, Wang Y, Qin X, Liu Y, Gobejishvili L, Joshi-Barve S, Ayvaz T, Petrosino J, Kong M, Barker D, McClain C, Barve S (2013). Metagenomic Analyses of Alcohol Induced Pathogenic Alterations in the Intestinal Microbiome and the Effect of Lactobacillus rhamnosus GG Treatment. PLoS One.

[45] Jabbour Z, do Nascimento C, Kotake BG, El-Hakim M, Henderson JE, de Albuquerque Junior RF (2013). Assessing the oral microbiome of healthy and alcohol-treated rats using whole-genome DNA probes from human bacteria. Arch Oral Biol.

[46] Walker AW, Sanderson JD, Churcher C, Parkes GC, Hudspith BN, Rayment N, Brostoff J, Parkhill J, Dougan G, Petrovska L (2011). High-throughput clone library analysis of the mucosa-associated microbiota reveals dysbiosis and differences between inflamed and non-inflamed regions of the intestine in inflammatory bowel disease. BMC Microbiol.

[47] Ismail IH, Oppedisano F, Joseph SJ, Boyle RJ, Licciardi PV, Robins-Browne RM, Tang ML (2012). Reduced gut microbial diversity in early life is associated with later development of eczema but not atopy in high-risk infants. Pediatr Allergy Immunol.

[48] Manichanh C, Rigottier-Gois L, Bonnaud E, Gloux K, Pelletier E, Frangeul L, Nalin R, Jarrin C, Chardon P, Marteau P, Roca J, Dore J (2006). Reduced diversity of faecal microbiota in Crohn’s disease revealed by a metagenomic approach. Gut.

[49] Le Chatelier E, Nielsen T, Qin J, Prifti E, Hildebrand F, Falony G, Almeida M, Arumugam M, Batto JM, Kennedy S, Leonard P, Li J, Burgdorf K, Grarup N, Jørgensen T, Brandslund I, Nielsen HB, Juncker AS, Bertalan M, Levenez F, Pons N, Rasmussen S, Sunagawa S, Tap J, Tims S, Zoetendal EG, Brunak S, Clément K, Doré J, Kleerebezem M (2013). Richness of human gut microbiome correlates with metabolic markers. Nature.

[50] Chang JY, Antonopoulos DA, Kalra A, Tonelli A, Khalife WT, Schmidt TM, Young VB (2008). Decreased diversity of the fecal Microbiome in recurrent Clostridium difficile-associated diarrhea. J Infect Dis.

[51] Hajishengallis G, Darveau RP, Curtis MA (2012). The keystone-pathogen hypothesis. Nature Reviews Microbiol.

[52] Lee J, Taneja V, Vassallo R (2012). Cigarette smoking and Inflammation: cellular and Molecular Mechanisms. J Dent Res.

[53] Brinig MM, Lepp PW, Ouverney CC, Armitage GC, Relman DA (2003). Prevalence of bacteria of division TM7 in human subgingival plaque and their association with disease. Appl Environ Microbiol.

[54] Podar M, Abulencia CB, Walcher M, Hutchison D, Zengler K, Garcia JA, Holland T, Cotton D, Hauser L, Keller M (2007). Targeted access to the genomes of low-abundance organisms in complex microbial communities. Appl Environ Microbiol.

[55] Zaura E, Keijser BJ, Huse SM, Crielaard W (2009). Defining the healthy “core microbiome” of oral microbial communities. BMC Microbiol.

[56] Liu BI, Faller LL, Klitgord N, Mazumdar V, Ghodsi M, Sommer DD, Gibbons TR, Treangen TJ, Chang YC, Li S, Stine OC, Hasturk H, Kasif S, Segrè D, Pop M, Amar S (2012). Deep sequencing of the oral microbiome reveals signatures of periodontal disease. PLoS One.

[57] Benítez-Páez A, Belda-Ferre P, Simón-Soro A, Mira A (2014). Microbiota diversity and gene expression dynamics in human oral biofilms. BMC Genomics.

[58] Gong HL, Shi Y, Zhou L, Wu CP, Cao PY, Tao L, Xu C, Hou DS, Wang YZ (2013). The Composition of Microbiome in Larynx and the Throat Biodiversity between Laryngeal Squamous Cell Carcinoma Patients and Control Population. PLoS One.

[59] Charlson ES, Bittinger K, Haas AR, Fitzgerald AS, Frank I, Yadav A, Bushman FD, Collman RG (2011). Topographical continuity of bacterial populations in the healthy human respiratory tract. Am J Respir Crit Care Med.

[60] Brook I (2005). The role of bacterial interference in otitis, sinusitis and tonsillitis. Otolaryngol Head Neck Surg.

[61] Darveau RP (2010). Periodontitis: a polymicrobial disruption of host homeostasis. Nat Rev Microbiol.

[62] Socransky S, Haffajee AD, Cugini MA, Smith C, Jr K (1998). Microbial complexes in subgingival plaque. J Clin Periodontol.

[63] Shchipkova AY, Nagaraja HN, Kumar PS (ᅟ). Subgingival Microbial Profiles of Smokers with Periodontitis. J Dent Res.

[64] Kembel SW, Wu M, Eisen JA, Green JL (2012). Incorporating 16S gene copy number information improves estimates of microbial diversity and abundance. PLoS Comput Biol.

[65] Angly FE, Dennis PG, Skarshewski A, Vanwonterghem I, Hugenholtz P, Tyson GW (2014). CopyRighter: a rapid tool for improving the accuracy of microbial community profiles through lineage-specific gene copy number correction. Microbiome.

[66] Adler CJ, Dobney K, Weyrich LS, Kaidonis J, Walker AW, Haak W, Bradshaw CJ, Townsend G, Sołtysiak A, Alt KW, Parkhill J, Cooper A (2013). Sequencing ancient calcified dental plaque shows changes in oral microbiota with dietary shifts of the Neolithic and Industrial revolutions. Nat Genetics.

