# Supplementary material for: Alcohol and tobacco consumption affects bacterial richness in oral cavity mucosa biofilms
Source: BMC Microbiol. 2014 Oct 3;14:250. doi: 10.1186/s12866-014-0250-2 (PMC4186948; doi:10.1186/s12866-014-0250-2)
Supplement: Additional file 1: Table S1. — Phyla log abundance. [file 12866_2014_250_MOESM1_ESM.doc]

Additional file 1: Supplementary Table 1. Phyla log abundance

| Phyla | Mean C | Mean S | Mean SD | P-Value | Rank | Adjusted P-Value |
| --- | --- | --- | --- | --- | --- | --- |
| *Acidobacteria* | 0.06387 | 0.00000 | 0.28546 | 0.16498 | 11 | 0.23997 |
| *Actinobacteria** | 4.64028 | 5.03887 | 4.70234 | 0.00101 | 2 | 0.00811 |
| *Armatimonadetes* | 0.09635 | 0.00000 | 0.00000 | 0.48567 | 15 | 0.51805 |
| *Bacteroidetes* | 3.19049 | 3.55231 | 3.08784 | 0.08847 | 9 | 0.15728 |
| *BD1-5** | 1.39643 | 0.00000 | 0.08637 | 0.01564 | 6 | 0.04170 |
| *TM7** | 2.42885 | 1.31464 | 2.25261 | 0.01259 | 5 | 0.04028 |
| *Chloroflexi* | 0.57954 | 1.23942 | 0.42868 | 0.05806 | 8 | 0.11611 |
| *Cyanobacteria** | 1.57230 | 0.00000 | 1.47512 | 0.00148 | 3 | 0.00788 |
| *Deinococcus-Thermus* | 0.48871 | 0.19336 | 0.26465 | 0.28942 | 12 | 0.38589 |
| *Firmicutes** | 4.94563 | 4.64525 | 4.94408 | 0.00511 | 4 | 0.02045 |
| *Fusobacteria** | 2.13600 | 2.75250 | 1.08493 | 0.00059 | 1 | 0.00948 |
| *Proteobacteria* | 4.46986 | 3.78472 | 4.09815 | 0.03049 | 7 | 0.06969 |
| *Spirochaetes* | 0.30773 | 0.19912 | 0.38493 | 0.49356 | 16 | 0.49356 |
| *Synergistetes* | 0.02375 | 0.50002 | 0.30261 | 0.14323 | 10 | 0.22917 |
| *Tenericutes* | 1.27203 | 0.75128 | 1.38967 | 0.37073 | 13 | 0.45628 |
| *TM6* | 0.12323 | 0.00000 | 0.00000 | 0.48567 | 14 | 0.55505 |

* - Indicates significantly altered phyla after multiple taxa correction.
