# Supplementary material for: Alcohol and tobacco consumption affects bacterial richness in oral cavity mucosa biofilms
Source: BMC Microbiol. 2014 Oct 3;14:250. doi: 10.1186/s12866-014-0250-2 (PMC4186948; doi:10.1186/s12866-014-0250-2)
Supplement: Additional file 2: Table S2. — Differential genera log abundance. [file 12866_2014_250_MOESM2_ESM.doc]

Additional file 2: Supplementary Table 2. Differential genera log abundance

| Genera | Mean C | Mean S | Mean SD | P-Value | Rank | Adjusted P-Value |
| --- | --- | --- | --- | --- | --- | --- |
| *Pedobacter* | 0 | 3.02732 | 0 | 0.00004 | 1 | 0.01076 |
| *Halocella* | 0 | 1.96889 | 0 | 0.00004 | 2 | 0.00538 |
| *Ralstonia* | 0.2065 | 2.32129 | 0.04864 | 0.00036 | 3 | 0.03356 |
| *Flavobacterium* | 0.31338 | 2.24589 | 0.03359 | 0.0005 | 4 | 0.03478 |
| *Peptostreptococcus* | 2.65827 | 0 | 1.59362 | 0.00084 | 5 | 0.04704 |
| *Gemella* | 2.8308 | 0.64093 | 3.20376 | 0.00091 | 6 | 0.04239 |
| *Abiotrophia* | 1.37293 | 0 | 0.38479 | 0.00093 | 7 | 0.03718 |
| *Thiomicrospira* | 0.92782 | 0 | 0.68199 | 0.00096 | 8 | 0.03353 |
| *Tannerella* | 0.29621 | 1.58955 | 0.06299 | 0.00112 | 9 | 0.03464 |
| *Kingella* | 0.79446 | 0 | 0 | 0.00117 | 10 | 0.03265 |
| *Achromobacter* | 0.16263 | 2.02721 | 0.28751 | 0.00126 | 11 | 0.03203 |
| *Oceanobacter* | 1.11139 | 0 | 0.15277 | 0.00127 | 12 | 0.02948 |
| *Microbacterium* | 1.2578 | 1.91293 | 0.38053 | 0.00128 | 13 | 0.02742 |
| *Prevotella* | 1.90205 | 3.15201 | 2.01229 | 0.00137 | 14 | 0.0273 |
| *Neisseria* | 3.93271 | 2.273 | 2.9324 | 0.00142 | 15 | 0.02644 |
| *Chloroplast* | 1.57068 | 0 | 1.47512 | 0.00153 | 16 | 0.02675 |
| *Stenotrophomonas* | 0.40916 | 2.78866 | 0.71105 | 0.00155 | 17 | 0.02551 |
| *Brachymonas* | 1.43152 | 0 | 0.41186 | 0.0016 | 18 | 0.02472 |
| *Staphylococcus* | 2.35495 | 0.12322 | 2.0939 | 0.00167 | 19 | 0.02454 |
| *Bergeyella* | 3.08142 | 0.63942 | 2.92323 | 0.00179 | 20 | 0.02497 |
| *Fusobacterium* | 1.63864 | 2.70878 | 0.94549 | 0.00191 | 21 | 0.02541 |
| *Psychromonas* | 0.85435 | 0 | 0.53169 | 0.00198 | 22 | 0.02515 |
| *Rikenellaceae; RC9_gut_group* | 0 | 0.72437 | 0 | 0.00214 | 23 | 0.02602 |
| *Elizabethkingia* | 0 | 0.75731 | 0 | 0.00214 | 24 | 0.02494 |
| *Sulfurovum* | 1.66416 | 0.10402 | 1.2403 | 0.00238 | 25 | 0.02652 |
| *Granulicatella* | 3.92744 | 3.31567 | 4.19676 | 0.00291 | 26 | 0.03118 |
| *Sphingomonas* | 0.29123 | 1.17536 | 0.09428 | 0.00295 | 27 | 0.03048 |
| *Arcanobacterium* | 0.53851 | 1.4009 | 0.5176 | 0.00314 | 28 | 0.03127 |
| *Rubrobacter* | 0.64616 | 0.12669 | 0.03917 | 0.00349 | 29 | 0.03353 |
| *Lachnospiraceae; Incertae_Sedis* | 1.59364 | 0.23423 | 0.83744 | 0.00379 | 30 | 0.03526 |
| *Bacillus* | 1.49661 | 0 | 1.04574 | 0.00433 | 31 | 0.03901 |
| *Oribacterium* | 3.16172 | 1.94023 | 2.96237 | 0.00453 | 32 | 0.03945 |
| *Atopostipes* | 0.9813 | 0 | 0.81339 | 0.00479 | 33 | 0.04053 |
| *Rhodanobacter* | 1.03821 | 0 | 0.18161 | 0.00512 | 34 | 0.042 |
| *Mogibacterium* | 3.20143 | 1.04317 | 2.74625 | 0.00558 | 35 | 0.04449 |
| *Shewanella* | 1.43482 | 0.34014 | 1.34574 | 0.00635 | 36 | 0.04922 |
| *Arthrobacter* | 1.93752 | 2.61169 | 1.90298 | 0.00652 | 37 | 0.04916 |
| *Capnocytophaga* | 0 | 0.69072 | 0.04902 | 0.00683 | 38 | 0.05018 |
| *Halothermothrix* | 0 | 1.0496 | 0.05526 | 0.00683 | 39 | 0.04889 |
| *Alcaligenes* | 0 | 0.90645 | 0.114 | 0.00683 | 40 | 0.04767 |
| *Clostridiales; Incertae_Sedis* | 3.20252 | 1.42411 | 2.30553 | 0.00704 | 41 | 0.0479 |
| *Peptoniphilus* | 0.40698 | 0 | 1.26604 | 0.00707 | 42 | 0.04694 |
| *Moryella* | 3.09589 | 1.36356 | 2.74679 | 0.00708 | 43 | 0.04596 |
| *Leifsonia* | 0.0183 | 0.84195 | 0 | 0.00723 | 44 | 0.04585 |
| *Variovorax* | 0.02197 | 1.07827 | 0 | 0.00723 | 45 | 0.04483 |
| *Aggregatibacter* | 2.43888 | 1.67022 | 1.34966 | 0.00728 | 46 | 0.04417 |
| *Agreia* | 0.47631 | 1.83782 | 1.27275 | 0.00795 | 47 | 0.04721 |
